# Highlight: Comparative Population Genomics—Answering Old Questions with New Data

**DOI:** 10.1093/gbe/evab278

**Published:** 2022-01-06

**Authors:** Casey McGrath

The fundamental principles of population genetics—including the role of mutation, selection, migration, and genetic drift—were established by evolutionary biologists such as Sewall Wright, J. B. S. Haldane, and Ronald Fisher in the first few decades of the 20th century. Nearly a century later, the field of population genomics leverages advances in genome sequencing, data storage, and bioinformatics to dissect the influence of these population-level forces at a genomic scale. At a higher level, comparative population genomics is now a growing field of study that involves investigating these forces at various taxonomic scales—from populations to species or even more distant groups—to understand the differences and commonalities in these patterns across lineages. These questions/topics are addressed in the Special Section titled “Comparative Population Genomics” and compiled by guest editors Tim Sackton, Director of Bioinformatics at Harvard University, and Russell Corbett-Detig, Assistant Professor of Biomolecular Engineering at the University of California Santa Cruz.

According to Sackton, this Special Section has been in the works for over a year. He and Corbett-Detig originally organized a symposium on Comparative Population Genomics for the 2020 Society for Molecular Biology and Evolution (SMBE) meeting that was canceled due to the coronavirus disease 2019 pandemic. Sackton notes that, with the encouragement of Editor-in-Chiefs Adam Eyre-Walker and Laura A. Katz, “we thought that a special section of *GBE* would be a good format to highlight some of the research in this emerging field that was not able to be presented” due to the cancelation.

The Special Section includes two review articles that help to put the emerging field of comparative population genomics into context. A review by Shoemaker et al. (2022) at the University of California Los Angeles highlights recent findings from the human gut microbiome, “a system with unparalleled potential for comparative population genomics studies” according to the authors. The article includes a reanalysis of patterns of purifying selection across approximately 40 species prevalent in the human gut microbiome, identifying functional categories that may be under more or less constraint in the microbiome.

The second review article discusses the link between the newer field of comparative population genomics and the much more established field of phylogeography. In an international partnership, Scott Edwards at Harvard University teamed up with collaborators from India, Portugal, and Australia to review the history of comparative phylogeography and its intersection with population genomics (Edwards et al. 2022). The authors argue that “comparative phylogeography offers an important perspective on evolutionary history that succeeds in integrating genomics with landscape evolution in ways that complement the suprageographic perspective of comparative population genomics.”

In addition to the above reviews, Sackton and Corbett-Detig “wanted to highlight some of the emerging areas of research that comparative population genetic data enables, especially as it becomes easier and easier to generate population variation datasets from many species.” For example, a study included in the Special Section by Goldberg and Harris (2022) from the University of Washington looks at mutational signatures from across the great ape phylogeny. Using whole-genome variation data from 88 great apes, Goldberg and Harris find that mutation spectrum evolution is largely driven by “trans-acting mutational modifiers that affect mutagenesis across the whole genome fairly uniformly.”

In another study, again led by Scott Edwards, a team investigated cryptic speciation and adaptive divergence in Bicknell’s and gray-cheeked thrushes (Termignoni-Garcia et al. 2022). These two bird species are difficult to distinguish in nature, exhibiting only minor differences in song and coloration. Using newly assembled reference genomes for each species as well as sequenced population samples, Termignoni-Garcia and colleagues confirm the genetic differentiation and low gene flow between the two species, while also suggesting a more recent split between the two lineages than what had been previously inferred. The researchers also identify potential mechanisms driving and/or reinforcing this divergence, including transposable element proliferation, rapid evolution of the Z-chromosome, and positive selection acting on genes involved in neuronal processes in Bicknell’s thrush.

Guest editors Sackton and Corbett-Detig also sought to highlight new approaches for analyzing comparative population genomic data sets in the Special Section. A multinational team of researchers from China, Denmark, France, and Sweden led by Martin Lascoux investigates how the distribution of fitness effects (DFE) of new mutations, a key parameter of molecular evolution (Chen et al. 2022) varies according to how conserved the site is in other species. This can help to characterize the DFE of beneficial mutations, a parameter that is notoriously difficult to estimate due to the rarity of these types of mutations.

Together, the articles in the Special Section highlight new findings, as well as new approaches and avenues for exploration in comparative population genomics. In addition, they show how these approaches can be used to link this emerging field to older disciplines that have historically relied on data from more limited markers. According to Sackton, this was a major motivation behind the Special Section: “I hope that highlighting the emerging power of comparative analysis of multispecies population variation datasets will stimulate new ideas in the field and encourage a growing emphasis on multispecies comparisons in population genetics.”
